# The Effects of Comorbidities on Outcomes After Total Hip Replacement

**DOI:** 10.3390/life16020194

**Published:** 2026-01-23

**Authors:** Hou Hoi Iong, Chih-Hung Chang, Jwo-Luen Pao, Wen-Chih Chen, Shang-Ming Lin, Cheng-Tzu Wang

**Affiliations:** 1 Department of Orthopedic, Far Eastern Memorial Hospital, New Taipei City 10617, Taiwan; 2 Department of Materials and Textiles, Asia Eastern University of Science and Technology, New Taipei City 10617, Taiwan; 3 Department of Mechanical Engineering, Asia Eastern University of Science and Technology, New Taipei City 10617, Taiwan

**Keywords:** total hip replacement, oxford hip score, patient-reported outcome, comorbidities, ASA physical status

## Abstract

Background: The relationship between comorbidity burden, as measured by the American Society of Anesthesiologists (ASA) classification, and functional recovery after total hip replacement (THR) remains uncertain. This study aimed to clarify whether ASA grade independently predicts postoperative patient-reported outcomes. Methods: We conducted a retrospective analysis of 218 consecutive patients from a prospectively maintained institutional registry who underwent primary unilateral THR between March 2021 and March 2024 in a single center. Patients were stratified into ASA 1–2 and ASA 3 groups. The Oxford Hip Score (OHS, 0–48) was collected preoperatively and at 1 week, 3 months, and 6 months postoperatively. Between-group differences were assessed, and multivariable linear regression was used to identify predictors of 6-month OHS. Results: Compared with ASA 1–2 patients, ASA 3 patients had lower preoperative OHS and longer hospital stay, but both groups showed substantial improvement over time and achieved excellent mean OHS at 6 months. In the adjusted model, higher ASA grade remained an independent negative predictor of 6-month OHS, whereas higher preoperative OHS and BMI were positive predictors. Conclusions: Despite presenting with worse baseline function and requiring longer hospitalization, ASA 3 patients experienced clinically meaningful recovery and achieved favorable 6-month outcomes after THR. Higher ASA status should therefore inform perioperative optimization rather than preclude surgery.

## 1. Introduction

Total hip replacement (THR) is a widely performed and highly effective procedure for end-stage osteoarthritis, offering significant pain relief and functional improvement [1,2,3]. Approximately 2.5 million individuals undergo THR in the U.S. each year [4]. As surgical volumes continue to increase worldwide, identifying patient-specific factors that influence postoperative recovery has become increasingly important for perioperative planning and shared decision-making.

The American Society of Anesthesiologists (ASA) Physical Status Classification is widely used as a practical and reproducible indicator of overall comorbidity burden and perioperative risk. The ASA physical status classification system, initially designed for general surgical procedures, has proven to be a valuable tool for assessing medical outcomes in major orthopedic surgeries, such as total hip replacement [5]. It provides clinicians with crucial information for preoperative discussions regarding patient outcomes [6]. While higher ASA grades have consistently been associated with increased complication rates and longer hospitalization, the relationship between ASA class and functional recovery is less clear. Several registry-based studies have suggested a stepwise decline in postoperative patient-reported outcomes with increasing ASA class [7]. Other reports indicate that although high-ASA patients present with worse preoperative function, they may achieve comparable long-term improvements after THR [8,9]. These heterogeneous findings highlight an ongoing uncertainty regarding the extent to which ASA independently predicts functional outcomes after accounting for baseline status.

The Oxford Hip Score (OHS) is a widely used patient-reported outcome (PRO) measure for evaluating pain, functional status, and recovery after total hip replacement (THR) [10]. It provides a standardized and patient-centered assessment of postoperative improvement and serves as an essential metric in contemporary arthroplasty registries and clinical research [11].

Given the existing variation in reported associations between ASA class and postoperative functional recovery, further clarification is needed, particularly in real-world clinical cohorts with prospectively collected PRO data. Therefore, this study aims to evaluate the relationship between ASA classification, recovery trajectories, and functional outcomes following THR. By analyzing OHS at multiple postoperative time points and adjusting for important baseline variables, this study seeks to determine whether ASA serves as an independent predictor of patient-reported functional recovery and to better define its clinical relevance in perioperative counseling and resource planning.

## 2. Materials and Methods

### 2.1. Study Design and Participants

Far Eastern Memorial Hospital is one of the twenty-two medical centers in Taiwan. In 2021, the Orthopedic Department established a clinical registry to systematically collect health outcomes before and after elective primary total hip replacement. Patients indicated for surgery were routinely measured for Oxford Hip Score in the outpatient department during pre- and post-surgery followup. Patients provided written consent to be included in the registry database. Inclusion criteria were adult patients (≥18 years old) undergoing unilateral primary THR for primary osteoarthritis of the hip with complete preoperative and postoperative OHS data. Patients were excluded if they had revision, bilateral, or staged THR surgeries. In the study of benefits, patients with missing OHS assessments or incomplete demographic or clinical data were also excluded. ASA 4 and 5 patients were not included because elective primary THR is not performed in such high-risk individuals at our institution; therefore, no ASA 4–5 cases were present in the registry during the study period. Although elements of enhanced recovery pathways were applied at the discretion of individual clinicians, no formal or standardized ERAS protocol was implemented during the study period. This observational study presents data from consecutive patients who underwent primary THR between March 2021 and March 2024. This study was a retrospective analysis of a prospectively maintained clinical registry, no a priori sample size or power calculation was performed, and the sample size was determined by the number of eligible patients available during the study period.

### 2.2. Data Collection

The ASA rating was assigned by the anesthetist and documented on the pre-anesthetic assessment data sheet. Health outcomes in total hip replacement were measured with the OHS. The OHS contains 12 questions that assess a patient’s hip function, pain, and ability to perform daily activities [12]. We used the adapted scoring system of Murray, where 48 points represents the best possible score and 0 points represents the worst possible score [13]. All assessments, including the 1-week assessment, were completed in the outpatient setting.

In addition to ASA grade and OHS, the registry systematically collected demographic and clinical variables including age, sex, body mass index (BMI), and length of hospital stay. These variables were extracted from the electronic medical record. A detailed description of the Oxford Hip Score and the American Society of Anesthesiologists physical status classification is provided in the Supplementary Materials (Table S1).

### 2.3. Outcome Measure

The primary outcome was the Oxford Hip Score at both preoperative and postoperative time points. According to the Kalairajah classification, OHS are interpreted as excellent (>41), good (34–41), fair (27–33), or poor (<27) [14,15]. The OHS was collected at standardized intervals (preoperatively, 1 week, 3 months, and 6 months postoperatively). LOS was determined from admission and discharge dates. The 1-week postoperative OHS was included to describe early postoperative recovery trajectories but was not intended to represent a definitive functional outcome measure.

### 2.4. Statistical Analysis

Descriptive data are presented as means ± standard deviations (SD) or percentages. To assess postoperative functional outcomes, uncorrected OHSs at pre-surgery, 1 week, 3 months, and 6 months postoperatively were compared between the low-comorbidity group (ASA 1–2) and high-comorbidity group (ASA 3) using independent t-tests. Comparisons between independent groups were performed using independent-samples t tests. Given the modest skewness of LOS, the results are presented using both mean ± standard deviation and median with interquartile range. Comparisons at individual postoperative time points were performed for descriptive purposes, and no formal longitudinal modeling was applied. The statistical significance threshold was set at *p* < 0.05. Absolute functional outcomes were analyzed to evaluate recovery trends over time. A multiple variable linear regression analysis was performed to investigate the effect of ASA grade (controlling for sex, pre-surgery OHS, BMI, and age) on 6-month post-surgery OHS. Collinearity among predictors was assessed using variance inflation factors (VIFs) and tolerance statistics. A correlation matrix among covariates was also examined. ASA grade was entered as an ordinal predictor representing increasing comorbidity burden. Repeated OHS measurements were used descriptively to illustrate recovery trends over time; inferential analyses focused on the six-month outcome, which was considered the primary clinically relevant endpoint. In response to concerns regarding model specification, ASA grade was additionally modeled as a dichotomous dummy variable (ASA 3 vs. ASA 1–2) in a sensitivity analysis to avoid assuming a linear dose–response relationship. All statistical tests were two-tailed, and a *p*-value < 0.05 was considered statistically significant. The results were reported as beta coefficients with 95% confidence intervals and *p*-values. All analyses were performed using statistical software SPSS, version 29.0. Normality of continuous variables was assessed using the Shapiro–Wilk test. All continuous variables demonstrated approximate normal distributions and were therefore summarized as mean ± standard deviation and analyzed using parametric tests. Changes in OHS over time within the same patients were evaluated using the repeated-measures Friedman test.

## 3. Results

**Figure 1:**
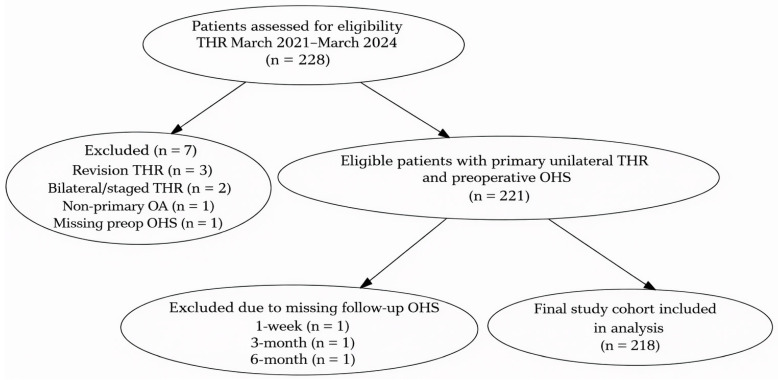
Flow diagram of patient selection.

Of the 228 patients who underwent total hip replacement during the study period, 7 were excluded based on predefined criteria and 3 were excluded due to missing follow-up OHS data. The final study cohort therefore comprised 218 patients with complete outcome data. The patient selection process is summarized in Figure 1. Among the 218 included patients, 5 (2.3%) were classified as ASA 1, 149 (68.3%) as ASA 2, and 64 (29.4%) as ASA 3. Patient characteristics for primary total hip replacement surgery are summarized in Table 1. The database included 218 unique patients who underwent primary total hip replacement between 2021 and 2024. The cohort comprised 98 men (44.9%) and 120 women (55.1%). The average age of patients who underwent hip replacement was 64.4 years (SD: 12.3). The mean preoperative OHS was 22.2 (SD: 10.7). At 1 week post operation, the mean OHS improved to 26.2 (SD: 8.7), followed by a substantial increase to 37.3 (SD: 6.7) at 3 months and further improvement to 43.3 (SD: 6.6) at 6 months. The OHS changed significantly over time in the overall cohort (Friedman test, *p* < 0.001).

Uncorrected scores for the OHS are presented in Table 2. Each patient had four measurement points: pre-surgery, 1 week post surgery, 3 months post surgery, and 6 months post surgery.

The mean preoperative OHS for the low-comorbidity group (ASA 1–2) and high-comorbidity group (ASA 3) was 23.5 (SD: 10.3) and 18.7 (SD: 11.0), respectively, with the high-comorbidity group having a significantly lower mean preoperative OHS (*p* = 0.003). At 3 months post surgery, the mean OHS was significantly higher in the low-comorbidity group with scores of 38.0 (SD: 6.6) compared to 35.6 (SD: 6.8) in the high-comorbidity group (*p* = 0.012). High-comorbidity group (ASA 3) was independently associated with a slightly lower 6-month uncorrected mean OHS. However, there was no statistically significant difference between the low-comorbidity group (ASA 1–2) and high-comorbidity group (ASA 3) at 1 week post surgery (26.7 ± 8.7 vs. 25.0 ± 8.7, *p* = 0.1) and 6 months post surgery (43.5 ± 4.4 vs. 42.7 ± 4.9, *p* = 0.14). The high-comorbidity group also had a significantly longer hospital stay, averaging 6 days (SD: 1.7), compared to 5.2 days (SD: 1.2) in the low-comorbidity group (*p* < 0.01). Length of stay showed a mildly right-skewed distribution. The median IQR LOS was 5 [4,5,6] days in the ASA 1–2 group and 6 [5,6,7] days in the ASA 3 group.

**Figure 2:**
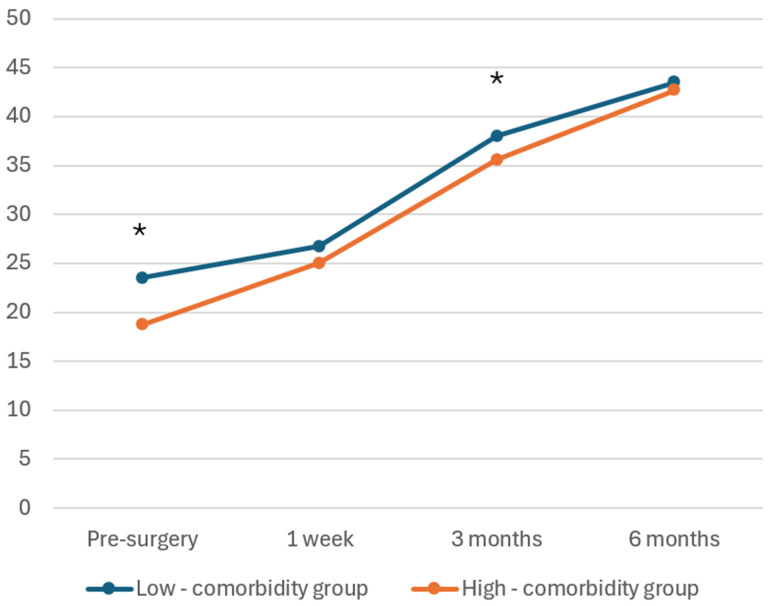
Uncorrected mean OHS for low-comorbidity and high-comorbidity groups in primary hip replacement. * *p* < 0.05.

The uncorrected mean OHSs for the low-comorbidity and high-comorbidity groups are presented in Figure 2. A steady increasing trend in OHSs was observed for both groups following surgery.

The results of the multivariable regression analysis evaluating the effect of ASA grade on 6-month post-surgery OHS are summarized in Table 3. The model demonstrated that a higher ASA grade was significantly associated with a lower 6-month OHS with a beta coefficient of −4.031 (95% CI: −6.018 to −2.044, *p* < 0.001), even after adjusting for baseline variables. Preoperative OHS was a positive predictor of 6-month OHS (B = 0.130, 95% CI: 0.047 to 0.213, *p* = 0.002), as was BMI with a beta coefficient of 0.226 (95% CI: 0.018 to 0.434; *p* = 0.033). Age and sex were not significantly associated with 6-month OHS (*p* = 0.186 and *p* = 0.845, respectively). When ASA was modeled as a dichotomous variable (ASA 3 vs. ASA 1–2), ASA 3 remained independently associated with lower six-month OHS, with an effect size comparable to the primary analysis, indicating that the observed association was primarily driven by ASA 3 status rather than a linear trend across ASA grades. Although the unadjusted six-month OHS did not differ significantly between ASA groups (Table 2), baseline OHS differed significantly between groups and was a strong predictor of postoperative OHS. Adjustment for baseline OHS reduced residual variance and clarified the independent association between ASA status and six-month outcomes. No evidence of problematic multicollinearity was observed (all VIFs ≤ 2.24).

## 4. Discussion

The principal finding of this study is that comorbidity burden, represented by the ASA physical status classification, demonstrated only a small but potentially clinically relevant decrement in six-month OHS after adjustment for baseline characteristics. Although patients with ASA 3 presented with significantly lower preoperative OHS, both ASA 1–2 and ASA 3 groups achieved excellent functional scores by six months, indicating that the overall recovery trajectory after THR remains highly favorable across comorbidity levels. These results are in line with international benchmarks, such as the New Zealand Joint Registry, which reported mean 6-month OHS values ranging from 35.2 to 42.1 across ASA classes in a cohort of over 22,000 patients undergoing primary THR [5].

When compared with previous research, our findings reinforce the view that THR provides substantial functional benefits even among patients with considerable comorbidity burdens. Studies by Berliner et al. (2015) and Husabø et al. (2016) similarly demonstrated that THR yields meaningful improvements in PROs across diverse patient groups, supporting the robustness of postoperative recovery in real-world settings [16]. Recent registry-based work has also highlighted that patient comorbidity profiles vary substantially across healthcare systems and may influence both baseline PROMs and postoperative recovery trajectories, underscoring the importance of appropriate case-mix adjustment when interpreting PROM comparisons [17]. In addition, contemporary observational studies have reported that higher multimorbidity burden is associated with less favorable patient-reported recovery and quality-of-life gains after hip replacement, and may also be linked to increased early postoperative complications and healthcare utilization [18]. Meta-analytic evidence using frailty-based constructs similarly demonstrates that higher preoperative vulnerability is associated with worse postoperative outcomes and greater complication risk following hip arthroplasty [19]. In our baseline-adjusted analysis, ASA grade exerted an independent effect on six-month OHS after controlling for baseline functional status. Importantly, the association persisted when ASA was modeled as a dichotomous variable, suggesting that ASA 3 status, rather than a graded linear increase across ASA classes, was the primary driver of the observed effect. It should be noted that the reported minimum clinically important difference (MCID) for the Oxford Hip Score was originally developed to reflect clinically meaningful within-patient change over time, rather than differences between independent groups. Therefore, although the adjusted between-group difference in six-month OHS approached the MCID range, this finding should be interpreted with caution when applied to between-group comparisons. In unadjusted analyses, ASA grade appeared to have a large and highly significant negative association with six-month OHS, largely reflecting substantial differences in baseline functional status between ASA groups. Because preoperative OHS is a dominant predictor of postoperative outcomes, adjustment for baseline OHS markedly attenuated the apparent effect of ASA grade, indicating that the strong unadjusted association was primarily driven by baseline differences rather than a direct effect on postoperative recovery [20]. Importantly, the residual between-group difference did not clearly exceed the reported minimum clinically important difference (MCID) threshold when interpreted in the context of adjusted between-group comparisons, suggesting limited clinical relevance despite statistical significance [11]. Consistent with this finding, the absence of a statistically significant difference in six-month OHS between patients with ASA 1–2 and ASA 3 indicates substantial functional overlap in unadjusted six-month outcomes, despite a persistent adjusted difference following total hip replacement [16].

Although long-term outcomes could not be directly evaluated in the present cohort, evidence from national and international joint registries has consistently demonstrated sustained functional improvement beyond one year following total hip replacement. Consistent with these findings, the functional improvements observed within both ASA groups from baseline exceeded the minimal clinically important difference (MCID) for OHS, estimated at 3–5 points based on the work of Murray et al. (2007) [13]. Their results, drawn from over 3000 patients, showed postoperative gains ranging from 11 to 27 points depending on baseline function, indicating that even patients with higher comorbidity can achieve clinically meaningful benefit. These findings show that high-comorbidity patients should not be excluded from surgical intervention. Nonetheless, consistent with prior evidence [21], the ASA 3 group experienced a slightly longer length of hospital stay, with a mean difference of less than one day. While this difference reached statistical significance, its absolute magnitude was modest. Importantly, this modest increase in hospitalization did not appear to offset the substantial functional gains achieved after surgery. Interventions such as prehabilitation, optimized discharge planning, and structured community-based follow-up may reduce LOS and overall healthcare burden while maintaining high-quality outcomes [22,23]. In addition to functional recovery and length of hospital stay, it is important to acknowledge that ASA classification has been consistently associated with perioperative complication risk in prior studies [5]. Patients with higher ASA grades have been shown to experience increased rates of medical and surgical complications following total hip replacement, which may influence early recovery trajectories and healthcare utilization. Because detailed complication data were not systematically captured in the present registry, this aspect could not be analyzed and represents an important limitation when interpreting the overall risk profile of high-comorbidity patients.

Our multivariable analysis further identified several important predictors of postoperative OHS. Preoperative OHS emerged as one of the strongest determinants of postoperative function, consistent with prior findings from Almeida-Ferrari et al. (2020), who reported that baseline OHS significantly predicted functional outcomes at both 6 and 12 months [20]. BMI demonstrated a small positive association with six-month OHS; however, the magnitude of this effect was minimal and unlikely to be clinically meaningful. Across the observed BMI range, the model-derived difference in predicted OHS was small. This finding should be interpreted with caution. Although higher BMI has not consistently been associated with superior outcomes in previous studies, the observed association in the present analysis may reflect residual confounding or unmeasured baseline characteristics, rather than a direct beneficial effect of BMI itself [20]. In contrast, age and sex did not significantly predict postoperative OHS, suggesting these demographic variables should not be considered barriers to receiving THR.

These results underscore the importance of incorporating comorbidity burden and baseline function into outcome prediction models. Rather than serving as a deterrent to surgery, ASA grade should be viewed as a risk-stratification variable that informs perioperative planning while recognizing that meaningful functional recovery remains achievable across comorbidity levels. Generalizability should be interpreted in the context of the study setting. This was a single-center study conducted at a tertiary medical center in Taiwan and primarily included patients undergoing elective primary total hip replacement for osteoarthritis. Differences in case mix, perioperative care pathways, rehabilitation availability, and healthcare reimbursement systems may influence postoperative recovery and length of hospital stay. In addition, cultural factors may affect the reporting of patient-reported outcome measures, including the Oxford Hip Score. Therefore, caution is warranted when extrapolating these findings to other populations, non-elective settings, revision procedures, or healthcare systems with differing perioperative protocols.

This study has several limitations. First, reliance on a single outcome measure may limit the comprehensiveness of recovery assessment. While OHS is a widely validated tool, incorporating other measures such as patient-reported quality of life and satisfaction could provide a more comprehensive understanding of recovery. In addition, although the Oxford Hip Score is a validated patient-reported outcome measure for assessing functional recovery after total hip replacement, the 1-week postoperative assessment is not a standard or formally validated timepoint for OHS and may be influenced by acute postoperative pain and in-hospital factors. Accordingly, results at this early timepoint should be interpreted with caution. Second, the regression model focused on patient-level baseline characteristics and did not include variables such as surgical technique, implant type, surgeon experience, or specific enhanced recovery protocols. Although these factors may influence postoperative outcomes, they were relatively standardized at our institution during the study period and were not consistently captured in the registry, necessitating a simplified modeling approach. Third, the follow-up duration in this study was limited to six months after surgery, which does not allow assessment of the durability of functional outcomes or long-term recovery trajectories following total hip replacement.

In addition, repeated Oxford Hip Score measurements were compared using independent tests at each time point, which does not account for within-subject correlation and may increase the risk of type I error due to multiple comparisons. A longitudinal analytical approach, such as a linear mixed-effects model, may provide a more robust assessment of recovery trajectories and should be considered in future studies. Furthermore, the ASA classification system, although widely used, serves as an aggregate indicator of overall comorbidity burden and does not capture the type or severity of individual comorbid conditions, nor postoperative complications, reoperations, or readmissions. In addition, its inherent subjectivity and potential interobserver variability may introduce bias [9,24]. The relatively small sample size, particularly for patients with specific comorbidities, may have limited the ability to detect meaningful associations with recovery, suggesting that future studies using larger datasets or national registries are warranted. Although no a priori sample size or power calculation was performed, the precision of the estimates is reflected in the relatively narrow 95% confidence intervals around the adjusted ASA effect, allowing exclusion of very small differences in six-month OHS. In addition, this study employed a complete-case analysis, and patients with missing OHS data at any follow-up time point were excluded. Although the proportion of excluded patients was small, this approach may introduce selection bias if patients with higher comorbidity burden or more complicated postoperative courses were less likely to complete follow-up assessments. Finally, surgeon preferences, subjective discharge decisions, and the non-randomized nature of patient selection may have contributed to variability in length of stay and limit the generalizability of the findings to broader hip replacement populations.

## 5. Conclusions

This study challenges the perception that patients with ASA 3 undergoing elective primary total hip replacement have poorer postoperative outcomes by demonstrating their significant recovery potential and favorable six-month functional results. While these patients required more intensive perioperative support and slightly longer hospital stays, their ability to achieve substantial functional improvement remained evident. These findings apply specifically to an elective ASA 1–3 primary THR population and should not be generalized to non-elective surgery, revision procedures, or patients with ASA 4–5.

## Figures and Tables

**Table 1:** Patient characteristics of primary total hip replacement.

Patient Characteristics	N (%)
Unique patient	218
Mean age (SD)	64.4 (12.3)
Age distribution	
18–30 years	2 (0.9%)
31–50 years	24 (11.0%)
51–75 years	159 (72.9%)
≥76	33 (15.1%)
Sex: male/female ASA classification distribution ASA 1 ASA 2 ASA 3	98 (44.9%)/120 (55.1%) 5 (2.3%) 149 (68.3%) 64 (29.4%)
Oxford Hip Score	
Mean pre-surgery (SD)	22.2 (10.7)
Mean post-surgery 1 week (SD)	26.2 (8.7)
Mean post-surgery 3 months (SD)	37.3 (6.7)
Mean post-surgery 6 months (SD) OHS over time (within-subject comparison)—*p* < 0.001 †	43.3 (6.6)

† *p*-value derived from repeated-measures Friedman test for within-subject comparison across the four time points (preoperative, 1 week, 3 months, 6 months postoperatively).

**Table 2:** Uncorrected values of the Oxford Hip Score and length of stay between ASA 1–2 and ASA 3. Values are presented as mean ± standard deviation. Sample sizes at each time point are shown in Table 1.

	Oxford Hip Score		
	ASA 1–2	ASA 3	
	Score (SD)	Score (SD)	*p*-Value
Pre-surgery	23.5 (10.3)	18.7 (11.0)	0.003
Post-surgery 1 week	26.7 (8.7)	25.0 (8.7)	0.100
Post-surgery 3 months	38.0 (6.6)	35.6 (6.8)	0.012
Post-surgery 6 months	43.5 (4.4)	42.7 (4.9)	0.140
Length of stay (LOS)	5.2 (1.2)	6 (1.7)	<0.010

**Table 3:** Output of multiple linear regression analysis to evaluate the effect of ASA grade on the 6-month OHS.

Variable	Coefficient	Std. Error	95% CI Lower	95% CI Upper	Std. Coeff.	t-Value	*p*-Value
Age (y)	−0.049	0.037	−0.121	0.024	−0.090	−1.327	0.186
BMI (kg/m^2^)	0.226	0.105	0.018	0.434	0.141	2.141	0.033
Pre-surgery OHS Sex (1 male, 2 female) ASA grade (1–3)	0.130 −0.202 −4.031	0.042 1.034 1.008	0.047 −2.241 −6.018	0.213 1.837 −2.044	0.209 −0.013 −0.270	3.081 −0.196 −3.999	0.002 0.845 <0.001

## Data Availability

The datasets used and/or analyzed in the current study are available from the corresponding author on reasonable request. Please contact the corresponding author. Dr. Cheng-Tzu Wang, Department of Orthopedic, Far Eastern Memorial Hospital, New Taipei City, Taiwan, e-mail: kingtzu1985@gmail.com.

## Supplementary Materials

The following supporting information can be downloaded at: https://www.mdpi.com/article/10.3390/life16020194/s1, Table S1.

## List of Abbreviations

THR	Total Hip Replacement
ASA	American Society of Anesthesiologists
OHS	Oxford Hip Score
LOS	Length of Stay
FEMH	Far Eastern Memorial Hospital
BMI	Body Mass Index
PRO	Patient-Reported Outcome
ERAS	Enhanced Recovery After Surgery
CI	Confidence Interval
SD	Standard Deviation
