# Supplementary material for: The Effects of Comorbidities on Outcomes After Total Hip Replacement
Source: Life (Basel). 2026 Jan 23;16(2):194. doi: 10.3390/life16020194 (PMC12941381; doi:10.3390/life16020194)
Supplement: Supplementary file 1 [file life-16-00194-s001.zip › life-4053299-supplementary.pdf]

Instrument	Description
**Oxford Hip Score (OHS)**	A patient-reported outcome measure consisting of 12 items assessing hip pain, function, and activities of daily living over the previous four weeks. Each item is scored on a five-point Likert scale, yielding a total score ranging from 0 to 48, with higher scores indicating better hip function. OHS score interpretation is commonly categorized as excellent (>41), good (34–41), fair (27–33), and poor (<27). The minimum clinically important difference (MCID) has been reported to range from 3 to 5 points.
**ASA Physical Status Classification**	A widely used preoperative risk stratification system that categorizes patients according to the presence and severity of systemic disease. ASA I indicates a normal healthy patient; ASA II, a patient with mild systemic disease; and ASA III, a patient with severe systemic disease that limits activity but is not incapacitating. Higher ASA grades have been associated with increased perioperative risk, complication rates, and healthcare utilization.
